# Results of Tendon Transfers in Radial Nerve Palsies: A New Evaluation Protocol

**DOI:** 10.3390/jpm14070758

**Published:** 2024-07-16

**Authors:** Micaela Reina, Simonetta Odella, Mauro Magnani, Francesco Locatelli, Alice Clemente, Martina Macrì, Pierluigi Tos

**Affiliations:** 1 Department of Orthopaedics and Traumatology, ASST Spedali Civili, 25123 Brescia, Italy; 2 Hand Surgery and Reconstructive Microsurgery Department, ASST Gaetano Pini-CTO, 20122 Milan, Italy

**Keywords:** radial nerve palsy, peripheral nerve injuries, tendon transfer, follow-up protocol

## Abstract

Radial nerve palsies present a challenging clinical scenario, often leading to substantial functional impairment. This study focuses on evaluating the outcomes of tendon transfer surgeries in patients with post-traumatic radial nerve injuries. The radial nerve, vital for upper limb movements, faces various etiologies, such as trauma, compression, or idiopathy. Patients with radial nerve palsy encounter difficulties in daily activities, emphasizing the need for effective management strategies. The research introduces a novel evaluation protocol, aiming to comprehensively assess tendon transfer outcomes. This protocol incorporates functional movements of wrist and finger joints, encompassing both objective and subjective parameters. The retrospective study includes eleven patients treated between 2010 and 2022, with a minimum follow-up of one year post-surgery. Tendon transfers demonstrated positive results. The evaluation protocol covers a wide range of parameters, including wrist and finger mobility, thumb function, grip strength, and patient satisfaction. The results indicate successful restoration of motor function, with an average grip strength of 70% compared to the healthy arm. The proposed evaluation protocol facilitates standardized and reproducible assessment, minimizing subjective errors in clinical evaluations. Despite the study’s limitations, such as a relatively small sample size, the findings underscore the effectiveness of tendon transfers in treating radial nerve palsies. The introduced evaluation scheme provides a comprehensive and reproducible approach to assess outcomes, contributing to the global standardization of tendon transfer assessments in radial nerve injuries.

## 1. Introduction

Radial nerve palsies represent a complex clinical condition that can lead to significant functional limitations and disability for affected patients [1]. From a physiological perspective, the radial nerve plays a key role in the motor control of the upper limb. It provides extrinsic extensor function to the wrist and hand. The main muscles innervated by the radial nerve include the triceps brachii, brachioradialis, wrist extensors, finger and thumb extensors, anconeus, and supinator. Therefore, the radial nerve plays a crucial role in the extension of the elbow, wrist, and fingers, hindering the grasp and release of objects, and is responsible for most of the strength (with a significant reduction in hand grip strength by up to 77% [2]) and coordination required for a proper movement of the upper limbs [3].

In addition to motor innervation, the radial nerve is also responsible for cutaneous sensitivity at the posterior surface of the arm, forearm, and hand (on the radial side), enabling tactile perception and pain sensitivity.

The incidence of radial nerve palsy can vary depending on the etiology, with traumatic, compressive, or idiopathic causes that can impair nerve function [4].

Patients with this condition often have difficulty performing essential daily actions, such as grasping and lifting objects, significantly limiting their quality of life and ability to perform work activities [5].

The management of radial nerve palsy has been the subject of ongoing research and development in orthopedics and nerve surgery [6]. Among the various treatment options available, tendon transfers have proven to be an effective surgical strategy for restoring motor function in patients with radial nerve damage. Tendon transfer is a surgical procedure in which the tendon of a functioning muscle is moved from its original position to reattach it to a new position in order to restore impaired motor function [7].

The main goal of tendon transfers in radial nerve palsies is to restore the ability to extend the elbow, wrist, and fingers, allowing patients to perform essential daily activities and, thus, improving their quality of life. Some schemes have been developed for evaluating the results of tendon transfers in radial nerve palsy, the most logical and commonly used being the evaluation schemes that assess the range of active joint movements.

The primary aim of our study is to present a new evaluation protocol, an original scheme for tendon transfer outcomes based on functional movements of the wrist and finger joints that includes all variables to be evaluated in the patients’ outcome, considering objective and subjective parameters, with a unique, complete, reproducible, and reliable scheme that minimizes the examiner’s subjective error in the evaluation of all clinical cases.

## 2. Materials and Methods

This is a retrospective study of clinical evaluation in patients who underwent tendon transfer for post-traumatic radial nerve injury. The study included forty-five patients, twenty-nine men and sixteen women, treated at G. Pini and C.T.O. Microsurgery and Hand Surgery Unit in Milan and C.T.O. in Turin, between 2010 and 2022. Eighteen patients who had brachial plexus injury were excluded from the study. In these cases, as the paralysis affected more than one nerve, the sample would not have been homogeneous. Sixteen patients were also excluded because they escaped follow-up or because they had died. We, therefore, included eleven patients who had a pure radial nerve lesion, nine men and two women. The average age of the patients was thirty-six years (19–83 y).

In eight patients, the dominant arm was affected. In six patients, the lesion was at the level of the posterior interosseous nerve (PIN) as a result of the forearm fracture, thus involving a low paralysis. In the remaining five patients, the radial paralysis was high (before the ramification of radial nerve in the PIN and sensory branch) as a result of a humerus fracture. All patients were evaluated after a minimum follow-up of one year following the surgery, with an average follow-up of three years and six months. One senior surgeon performed all the operations.

In all cases, the same technique, described by Riordan [8], was used: flexor carpi ulnaris (FCU) pro common finger extensor (EDC), used to restore finger extension; pronator teres (PT) pro extensor radialis carpi brevis (ECRB) to restore wrist extension; and palmaris longus (PL) pro extensor pollicis longus (EPL) to restore thumb extension using the re-routing technique [9], in which the EPL is rerouted from Lister’s tubercle to the anatomical snuffbox to allow recovery of both thumb extension and abduction. The post-operative management was the same for all the patients.

### Surgical Technique

**Figure 1:**
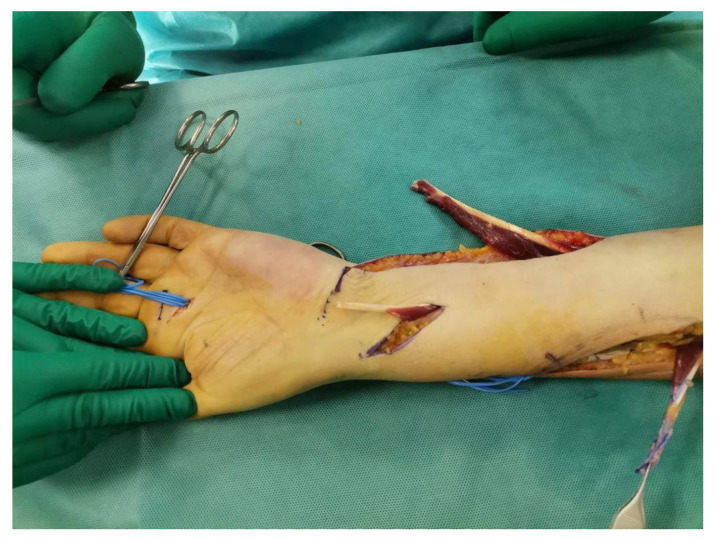
Example of surgical incision at the half and distal forearm with the three tendons ready to be transferred. In the ulnar side the FCU, in the center the PL, in the radial side the PT. The explanation is provided in the subsequent text.

The surgical technique involves plexus anesthesia with pneumo-ischemia of the arm. The first volar J-incision is made longitudinally at the FCU from the distal half of the forearm to the wrist and the distal transverse extension is extended to the PL. The FCU tendon is retrieved just proximal to the pisiform and released as far as the proximal extension of the incision allows. The FCU muscular belly is very wide and, therefore, it is preferred to remove part of the belly at the myotendinous insertion to facilitate later mobilization and avoid bulk around the ulnar edge of the forearm (Figure 1).

The second dorsal incision, of approximately 5 cm, is made 2 cm distal to the medial epicondyle and directed towards Lister’s tubercle. The deep fascia of the FCU and all fascial shoots are then removed in order to mobilize it properly. The third incision (Figure 1) begins in the volar radial aspect of the forearm and is directed dorsally around its radial border in the PT insertion region.

**Figure 2:**
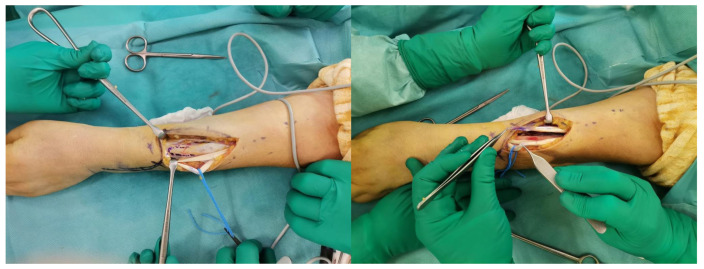
The image shows PT identification and sampling with a strip of periosteum.

The insertion on the radius of the PT is identified and disengaged, paying attention to take a strip of periosteum together to ensure that the length is sufficient (Figure 2). The PT is then transferred subcutaneously (above the BR and ECRL) and sutured according to the Pulvertaft technique [10], with 4-0 non-resorbable thread, just distal to the myotendinous junction of the ECRB, under maximum tension and with the wrist at 45° extension.

**Figure 3:**
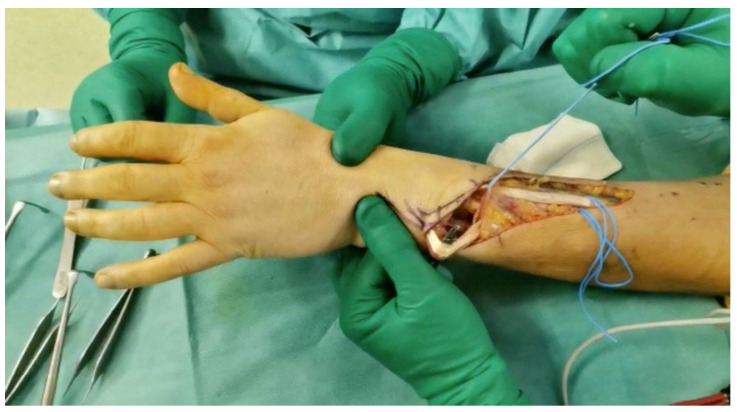
The picture shows FCU transferred proximal to the extensor retinaculum, where it is sutured with each tendon of EDC.

At this point, the FCU is transferred subcutaneously to the third incision, where it is sutured distal to the myotendinous junction at each tendon of the EDC, just proximal to the extensor retinaculum (Figure 3), with 4-0 non-absorbable thread, under maximum tension with the wrist in the neutral position. It is essential that the traction line is as straight as possible from the medial epicondyle to the EDC.

**Figure 4:**
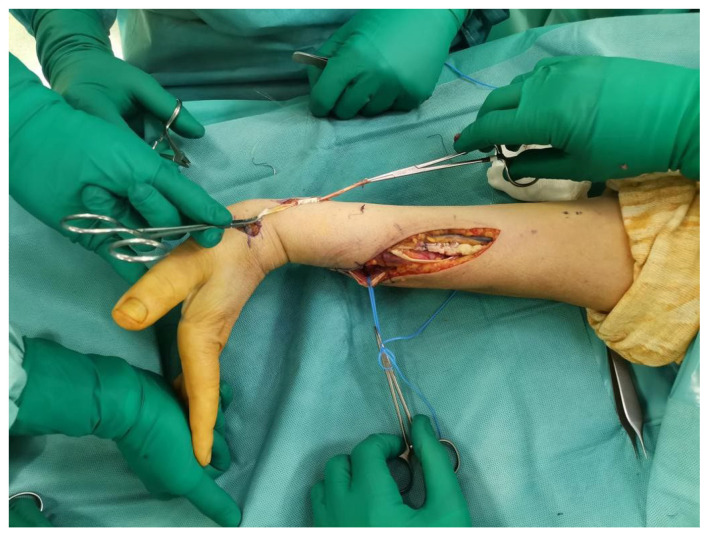
The picture shows EPL dissected and pulled out of the extensor retinaculum and redirected to have both extension and abduction of the thumb. The wrist is taken in extension after PT and then transferred to ERBC.

Through the third incision (Figure 1), the EPL is dissected at the myotendinous junction proximal to the extensor retinaculum; from here, it is pulled out of its channel and redirected through a subcutaneous tunnel, at the level of the anatomical snuffbox, to the I metacarpal and brought volarly [9]. The PL is disengaged at the wrist and sutured to the EPL, according to the Pulvertaft technique, with 4-0 non-absorbable thread in maximum tension at neutral wrist (Figure 4). The PL is absent in approximately 14% of the population [11,12]; in these cases, the EPL is integrated with the EDC, but the independence of the first finger is lost.

Before closing the wounds, tension should be tested by passively moving the wrist to show the synergic action of the new transfers. With the wrist in extension, fingers flexion into the palm should be possible and, with the wrist in flexion, full extension of the metacarpo-phalanges should be possible, but not hyperextension. In patients who presented low paralysis PT transfer, pro ECRB was not performed.

The post-operative protocol involves immobilization in a brachial–antebrachial–metacarpal (BAM) plaster cast for three weeks, with the proximal interphalangeal joints (PIP) free, the forearm pronated approximately 15°–30°, the wrist extended to 45°, the metacarpo-phalangeal (MP) joints slightly flexed to 10°–15°, and the thumb in full extension and abduction. After that, a short brace is applied for a further three weeks, which the patient can remove several times per day to begin the rehabilitation treatment. A good recovery of function is generally observed after three months. Maximum recovery generally occurs six months after surgery.

## 3. Results

All patients were evaluated after a minimum follow-up of one year after surgery, with an average follow-up of three years and six months. Measurements were performed with a goniometer. We used an original evaluation protocol that include all possible variables to be evaluated, specifically: wrist extension and flexion with flexed and extended fingers; radial deviation (RD) and ulnar deviation (UD) of the wrist; MP extension of the long fingers with flexed wrist and in neutral position; extension–abduction of the thumb by assessing the degrees of the trapezio-metacarpal (TM), MP, and interphalangeal (IP) joints; thumb flexion with the Kapandij test; and the flute test for finger independence and strength with Jamar’s dynamometer, compared with the non-affected hand. We also assessed the degree of personal satisfaction, time to return to work, and the DASH score (Table 1).

**Figure 5:**
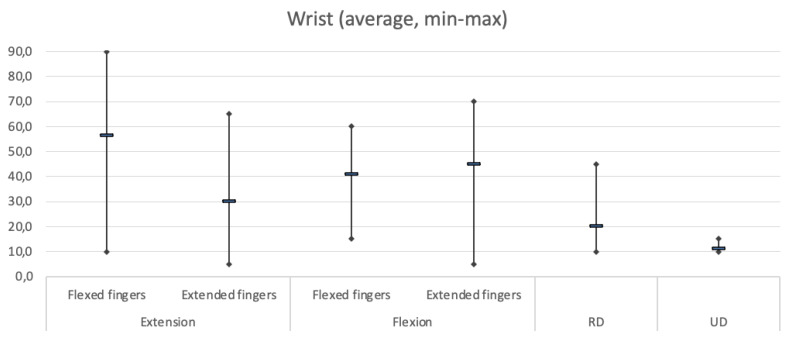

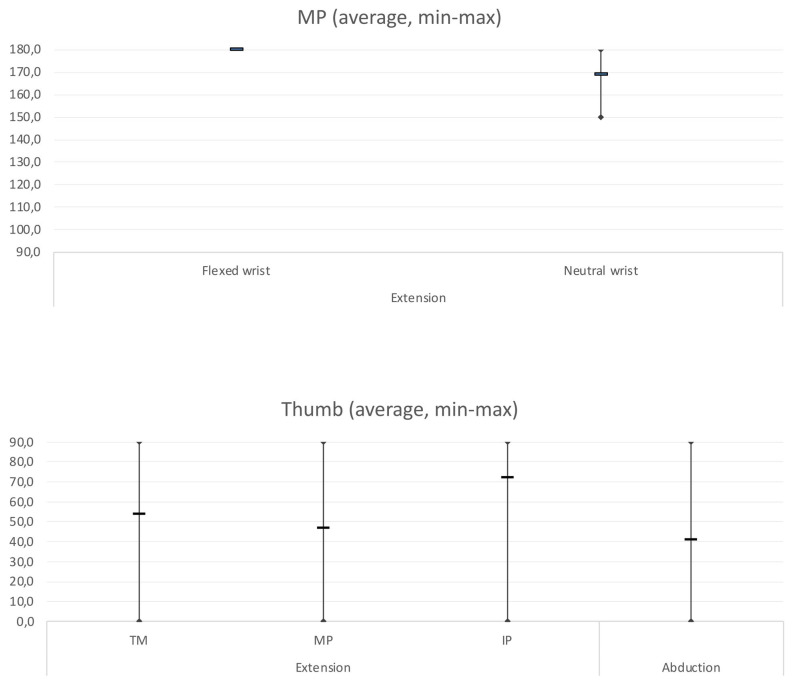
Shows average of wrist ROM (range of motion) degree with flexed and extended fingers and RD-UL, average of MP extension, and average of thumb extension–abduction.

**Figure 6:**
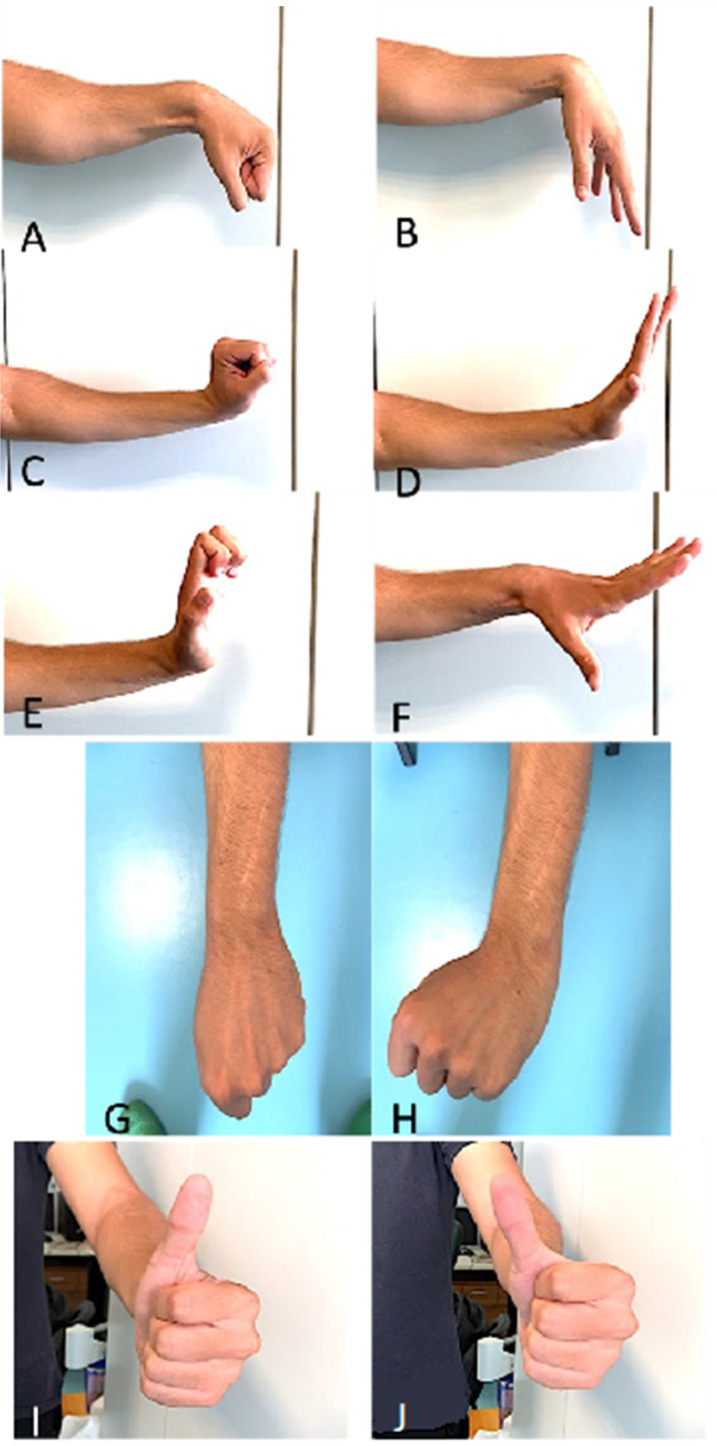
Clinical images of wrist flexion with flexed (**A**) and extended fingers (**B**), wrist extension with flexed (**C**) and extended fingers (**D**); MP extension with extended (**E**) and neutral wrist (**F**); UD (**G**) and RD (**H**); extension (**I**) and abduction (**J**)of the thumb.

As far as range of motion (ROM) is concerned, Table 2 and Figure 5 show in detail: the average degrees of movement obtained for each individual assessment, with minimum and maximum ranges and the standard deviation. Figure 6 shows examples of clinical results.

The average wrist extension angle was 56.3° when flexing the fingers and 30° when extending the fingers. The average wrist flexion angle was 40.9° when flexing the fingers and 45° when extending the fingers. The average wrist radial deviation was 20° and the ulnar deviation was 11°. All patients could fully extend their fingers when the wrist joint was flexed, while the average fingers extension was 169.1° the with wrist in neutral position.

The average thumb extension angle was 54° at the TM joint, 47° at the MP joint, and 72° at the IP joint. The average thumb abduction angle was 41°. The surgery did not induce wrist joint rotational deformity.

**Figure 7:**
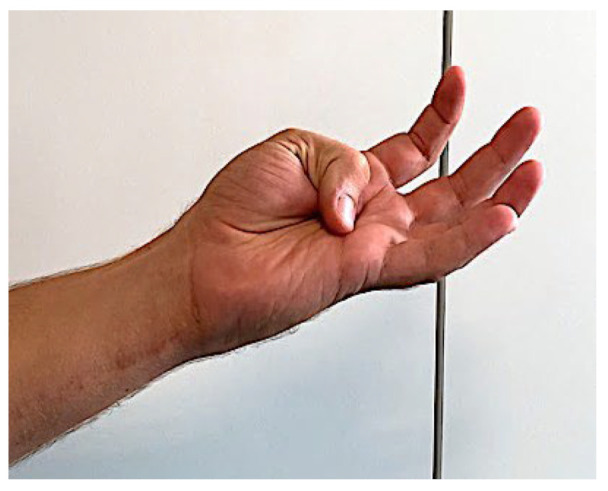
Picture shows a Kapandji test of 10.

The Kapandji test evaluation showed an average of 8.7, meaning that, on average, patients were able to touch the base of the fifth finger (Figure 7).

Grip strength was assessed using Jamar’s dynamometer and evaluated against the strength of the healthy arm. On average, the patients showed 70% of the contralateral strength. The DASH score showed an average value of 26.2%, with good patient-reported results and a minimal degree of disability. All patients were very satisfied and had no limitations in their daily activities, with return to work in all cases after an average period of seven months after surgery.

**Figure 8:**
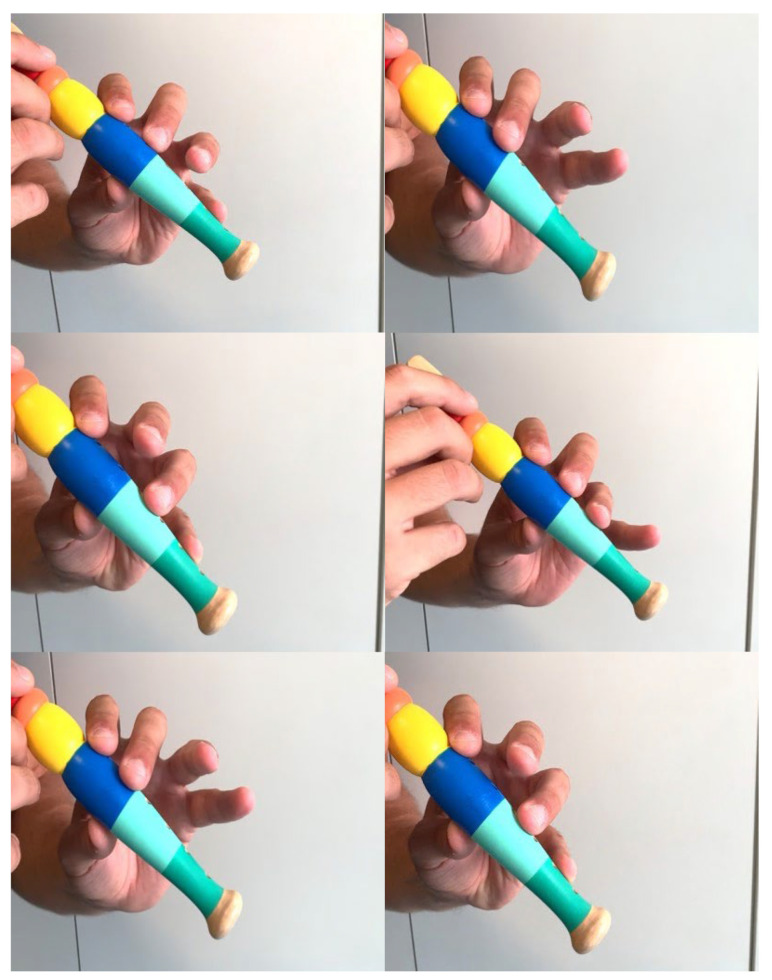
Flute test. Images show different degrees of fingers independence.

In three patients, we noticed a certain degree of finger independence by means of the flute test. Specifically, the patient was asked to ‘play the flute’ by pressing the holes with single and paired fingers with MP in extension. We found that the three patients performed more easily with a single finger, with II and IV fingers together, and with III and IV fingers together. This can be explained by the independence of the flexor tendons to overcome extensor resistance (Figure 8). Another hypothesis is that after tendon transfer, it takes several months for the cortical domain to adapt, resulting in a delay before improvements, such as those observed in the flute test, become evident. This phenomenon could be a subject of future studies.

There were no major complications, such as infection or suture rupture. One patient, aged 83, had a fall eight months after the operation, resulting in a wrist fracture, treated conservatively, on the same side as the tendon transfer surgery. This undoubtedly affected the current result, as she reported that before the new trauma, she had regained most of her functions, which were then lost due to the fracture, leading to stiffness in the wrist.

Analyzing these initial results using our rating scale, we can affirm that, on average, wrist extension was good with flexed fingers and excellent with extended fingers, wrist flexion was excellent with flexed fingers and good with extended fingers, showed excellent radial deviation and good ulnar deviation, and MP extension was excellent with flexed wrist and good with neutral wrist. As for the thumb, TM extension was good on average, MP extension was good and IP extension was excellent, abduction was satisfactory, and flexion was good (measured with the Kapandji test).

**Figure 9:**
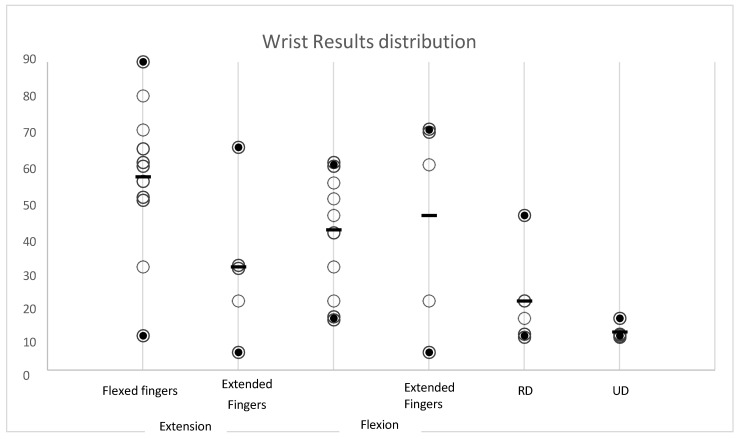
The final distribution graph of the wrist values.

We report in Table 3 the individual percentages and the final distribution graph of the wrist values (Figure 9).

## 4. Discussion

The radial nerve is frequently affected in upper extremity fractures, especially humerus fractures where the incidence reaches 12% [13,14]. Radial nerve injuries due to diaphyseal fracture of the humerus may be associated with Holstein–Lewis fractures: fractures of the middle/distal third of the humerus, where the radial nerve twists to move anteriorly and is exposed to stretching/pulling damage due to its position between two fibrous fixation means (proximally the intermuscular septum and distally the Frohse’s arch) [1,15]. Fractures of the proximal third of the radius are also at risk of radial nerve injury. Furthermore, a great risk is iatrogenic damage due to surgical access [16].

Because of its particular course, characterized by its close relationship with the humerus, the radial nerve has several sites of possible compression: the radial tunnel at the proximal forearm, Frohse’s arch (PIN), the passage between the brachioradialis muscle tendon and extensor longus carpi (dorsal sensory branch), and Wartenberg syndrome (compression of the radial sensory branch with only sensory symptoms) [17]. Rarely, chronic compressive syndromes of the radial nerve cause clinically relevant pictures of paralysis.

The most common clinical presentation of radial nerve palsies is characterized by denervation of the extensor musculature with the characteristic ‘Wrist-Drop Sign’ [18]. In complete paralysis, extension is maintained at the level of the interphalangeal joints, as it affects the intrinsic musculature innervated by the ulnar nerve.

Radial nerve injuries should be divided into high and low injuries depending on whether the level of injury is proximal or distal to the emergence of the PIN [19]. In high lesions, at the level of the humeral diaphyseal middle third, denervation of the brachioradialis muscle is also present. The area of hypo-anesthesia is the one pertaining to the superficial radial sensory nerve. In proximal paralysis (isolated proximal lesions related, for example, to iatrogenic damage during surgery in the proximal region of the humerus) or posterior plexus cord lesions, triceps denervation with elbow extension deficit is also present [3,4,6].

Preservation of wrist extension in radial deviation indicates a radial nerve injury at the level of the PIN (low injury), as the branch to the radial extensor muscle of the carpus is supplied prior to the entry into the fibrotic canal.

In most cases, the nerve does not suffer real injuries, but only a stretch or contusion. These are very often Sunderland grade I and II injuries [20], and this explains the high spontaneous recovery rate, from 70 to 90%, of these injuries. In such cases, recovery is gradual but complete [13,14,19]. Other times, however, we are faced with high-energy traumas that result in a complete nerve injury, with or without loss of substance. In these cases, it may be necessary to perform a tendon transfer later to restore the lost function [21]. The main indication for upper extremity tendon transfer is a peripheral nerve injury with no potential improvement [18,22,23].

The timing of tendon transfers can be classified as early, conventional, or delayed [24]. Brand [25] and Burkhalter [26] have recommended early tendon transfers under specific circumstances, suggesting that the procedure be carried out simultaneously with or prior to nerve repair. Early tendon transfer, thus, acts as a temporary substitute for the paralyzed muscle until reinnervation occurs, functioning like an internal splint. If reinnervation is suboptimal, early tendon transfer boosts the strength of the partially paralyzed muscle; if reinnervation does not occur, it serves as a permanent substitute. When the nerve gap exceeds 4 cm, or there are extensive wounds or significant skin loss over the nerve, performing tendon transfers immediately is appropriate due to the low likelihood of spontaneous recovery. Conversely, when there is no clear evidence of a nerve lesion, or a neurorrhaphy or nerve graft has been performed, it is advisable to wait. By considering a nerve regrowth rate of 1 mm per day, the time required to detect functional recovery clinically can be estimated by calculating the distance between the lesion and the muscular target innervation [4]. Conventionally, tendon transfer is performed twelve months after injury, because the paralyzed muscle is irreversibly damaged and there is no more recovery potential [27].

There are several common tendon transfer approaches used for the treatment of radial nerve palsies [4,7,19,24].

According to the literature, the wrist extension angles observed in our study are consistent with those reported by other authors who achieved wrist extension restoration by transferring the pronator teres tendon to the extensor carpi radialis brevis [28,29].

The optimal tendon transfer for finger function remains a debated topic in the scientific literature. Some authors prefer using the flexor carpi radialis for tendon transfers instead of the flexor carpi ulnaris, due to concerns that the latter may cause radial deviation of the wrist [28,30]. However, along with other authors [31,32], we prefer the flexor carpi ulnaris, except in the case of heavy manual laborers, such as masons, for whom ulnar deviation—an important movement necessary for activities like hammering and throwing—would also be lost. Therefore, the choice of tendon transfers is a matter of surgeon’s preference and there seems to be no difference in the final outcome [31].

The two most frequently used transfers today are the Brand [33] and Riordan transfers [8]. They both employ the palmaris longus (PL) to the re-routed extensor pollicis longus (EPL) to address thumb extension and radial abduction.

Although the literature is abundant with articles on tendon transfers, there are relatively few studies that have analyzed the results using evaluation score systems. The standardization of results in tendon transfers is not straightforward [18,34].

The heterogeneity of outcome classification remains a limitation in the evaluation of tendon transfer surgery. A recent review by Compton et al. [6] suggested that comparisons between techniques are not possible, precisely because of the heterogeneity in reporting outcomes.

The most frequently used scales are Bincaz’s [35] and Zachary’s [36]. Another classification used is the Tajima T’s classification [37]. In general, however, these systems classify the results into different groups without precise indicators, allowing for subjective interpretation and different endpoints in the same patient with different examiners. Furthermore, little attention is paid to the patient’s subjective functional results. Zachary’s scheme does not define the variables objectively and their variables are defined as “mild or severe” loss of function.

In a recent work, Karabeg et al. [17] developed a new evaluation system that, like ours, aims at standardization and repeatability of the system. With the present study, we have completed and supplemented the attempt to standardize the assessment of results in palliative radial nerve palsy surgery by introducing new factors, such as the flute test, which aims at assessing the recovery, albeit partial, of finger independence, as well as the strength recovered and the subjective component of patient satisfaction.

Our study presents some limitations, one being the small number of patients, although it was a homogeneous group.

## 5. Conclusions

Forearm tendon transfer is a crucial technique for restoring wrist, finger, and thumb extension function following irreparable damage to the radial nerve. Careful patient assessment and accurate planning of the surgery are mandatory to achieve positive results. Currently, no standardized surgical procedure for tendon transfer is universally recommended; each surgery must be tailored to meet the individual patient’s requirements. There is a pressing need to establish a comprehensive and universally accepted evaluation protocol for tendon transfer outcomes to facilitate meaningful comparisons across studies. A functional assessment scheme is essential for evaluating the outcomes of this kind of surgery. We propose, with our work, a new, original, simple, and reproducible evaluation protocol that includes all variables to be assessed in the outcome of tendon transfers of the radial nerve, taking into account objective parameters of active mobility of the wrist, finger, and thumb joints and subjective parameters. Such a protocol aims at the standardization of results in tendon transfers of radial nerve palsy outcomes that can be accepted worldwide, to simplify the assessment of diverse clinical outcomes across different variables and to facilitate comparative analysis of results.

## Figures and Tables

**Table 1:** Our new evaluation protocol that includes objective and subjective evaluation.

Degrees		Value	Excellent	Good	Satisfactory	Poor
WRIST (0°–90°)						
Extension	Flexed fingers		≥60°	30–60°	0–30°	<0°
	Extended fingers		≥30°	15–30°	0–15°	<0°
Flexion	Flexed fingers		≥30°	15–30°	0–15°	<0°
	Extended fingers		≥60°	30–60°	0–30°	<0°
Radial Deviation			≥20°	10–20°	0°–10°	Inability of DR
Ulnar Deviation			≥20°	10–20°	0°–10°	Inability of DU
MP (90°–180°)						
Extension	Flexed Wrist		≥170°	150–170°	130–150°	<130°
	Neutral Wrist		≥170°	150–170°	130–150°	<130°
Thumb (0°–90°)						
Extension	TM		≥70°	45–70°	30–45°	<30°
	MP		≥70°	45–70°	30–45°	<30°
	IP		≥70°	45–70°	30–45°	<30°
Abduction			≥70°	45–70°	30–45°	<30°
Flexion (Kapandji)			9–10	8	7	<7
Test		Value	Range			
Flute test (Fingers Independence)			Yes–No			
Degree of personal satisfaction			0–10			
Time to back to work			Months			
Grip strength			Jamar (percentage compared to the healthy side)			
Dash score			(0–100) https://orthotoolkit.com/dash/ (accessed on 20 April 2024).			

**Table 2:** Shows in detail: the average degrees of movement obtained for each individual assessment, with minimum and maximum ranges and the standard deviation.

Degrees		Average	Results		Std Dev
WRIST (0°–90°)			Min	Max	
Extension	Flexed fingers	56.3	10.0	90.0	22.1
	Extended fingers	30.0	5.0	65.0	22.1
Flexion	Flexed fingers	40.9	15.0	60.0	18.1
	Extended fingers	45.0	5.0	70.0	30.4
Radial Deviation		20.0	10.0	45.0	14.6
Ulnar Deviation		11.0	10.0	15.0	2.2
MP (90°–180°)					
Extension	Flexed Wrist	180.0	180.0	180.0	0.0
	Neutral Wrist	169.1	150.0	180.0	11.6
Thumb (0°–90°)					
Extension	TM	54.0	0.0	90.0	37.8
	MP	47.0	0.0	90.0	42.7
	IP	72.0	0.0	90.0	37.9
Abduction		41.0	0.0	90.0	32.5
Flexion (Kapandji)		8.7	8.0	10.0	0.8
Test					
Degree of personal satisfaction		9.4	9.0	10.0	0.5
Time to back to work		7.3	4.0	12.0	4.2
Grip strength		69.4%	30.0%	100.0%	0.2
Dash score		26.2	1.7	60.7	21.3

**Table 3:** Individual percentages of the wrist values.

Degrees		Excellent	Good	Satisfactory	Poor
WRIST (0°–90°)					
Extension	Flexed fingers	54.5%	36.4%	9.1%	0.0%
	Extended fingers	60.0%	20.0%	20.0%	0.0%
Flexion	Flexed fingers	72.7%	27.3%	0.0%	0.0%
	Extended fingers	60.0%	0.0%	40.0%	0.0%
Radial Deviation		40.0%	60.0%	0.0%	0.0%
Ulnar Deviation		0.0%	100.0%	0.0%	0.0%
MP (90°–180°)					
Extension	Flexed Wrist	100.0%	0.0%	0.0%	0.0%
	Neutral Wrist	54.5%	45.5%	0.0%	0.0%
Thumb (0°–90°)					
Extension	TM	60.0%	0.0%	20.0%	20.0%
	MP	40.0%	20.0%	0.0%	40.0%
	IP	80.0%	0.0%	0.0%	20.0%
Abduction		20.0%	20.0%	40.0%	20.0%
Flexion (Kapandji)		54.5%	45.5%	0.0%	0.0%

## Data Availability

The raw data supporting the conclusions of this article will be made available by the authors on request.
